# Pyrrolysyl-tRNA Synthetase with a Unique Architecture Enhances the Availability of Lysine Derivatives in Synthetic Genetic Codes

**DOI:** 10.3390/molecules23102460

**Published:** 2018-09-26

**Authors:** Atsushi Yamaguchi, Fumie Iraha, Kazumasa Ohtake, Kensaku Sakamoto

**Affiliations:** 1 Division of Structural and Synthetic Biology, RIKEN Center for Life Science Technologies (CLST), 1-7-22 Suehiro-cho, Tsurumi, Yokohama 230-0045, Japan; firaha@ab.cyberhome.ne.jp (F.I.); kazumasa.ohtake@riken.jp (K.O.); 2 Laboratory for Nonnatural Amino Acid Technology, RIKEN Center for Biosystems Dynamics Research (BDR), 1-7-22 Suehiro-cho, Tsurumi, Yokohama 230-0045, Japan

**Keywords:** genetic code expansion, amber suppression, archaea, benzyloxycarbonyllysine, protein engineering

## Abstract

Genetic code expansion has largely relied on two types of the tRNA—aminoacyl-tRNA synthetase pairs. One involves pyrrolysyl-tRNA synthetase (PylRS), which is used to incorporate various lysine derivatives into proteins. The widely used PylRS from Methanosarcinaceae comprises two distinct domains while the bacterial molecules consist of two separate polypeptides. The recently identified PylRS from *Candidatus* Methanomethylophilus alvus (CMaPylRS) is a single-domain, one-polypeptide enzyme that belongs to a third category. In the present study, we showed that the PylRS—tRNA^Pyl^ pair from *C.* M. alvus can incorporate lysine derivatives much more efficiently (up to 14-times) than Methanosarcinaceae PylRSs in *Escherichia coli* cell-based and cell-free systems. Then we investigated the tRNA and amino-acid recognition by CMaPylRS. The cognate tRNA^Pyl^ has two structural idiosyncrasies: no connecting nucleotide between the acceptor and D stems and an additional nucleotide in the anticodon stem and it was found that these features are hardly recognized by CMaPylRS. Lastly, the Tyr126Ala and Met129Leu substitutions at the amino-acid binding pocket were shown to allow CMaPylRS to recognize various derivatives of the bulky *N*^ε^-benzyloxycarbonyl-l-lysine (ZLys). With the high incorporation efficiency and the amenability to engineering, CMaPylRS would enhance the availability of lysine derivatives in expanded codes.

## 1. Introduction

Pyrrolysyl-tRNA synthetase has been applied for expanding the repertoire of genetically encoded amino acids [1,2,3,4]. PylRS naturally occurs in archaea and certain bacteria species attaching pyrrolysine to tRNA^Pyl^ to translate the UAG amber stop codon [1,5]. The utility of PylRS is based on the following observations. First, this pair does not cross-react with the aminoacyl-tRNA synthetase (aaRS)—tRNA pairs of host cells such as *Escherichia coli* and mammalian cells. The noncanonical secondary structure of tRNA^Pyl^, which is a small D loop and an extended anticodon stem, apparently underlines this orthogonality [6]. Second, since pyrrolysine is not included in the metabolic pathways of host cells, the promiscuous incorporation of pyrrolysine and its derivatives into proteins is avoided even when PylRS variants are not strictly specific for the derivatives and retains the affinity for the parent amino acid. This advantage greatly facilitates PylRS engineering. Lastly, the tRNA^Pyl^ recognition by PylRS does not involve the anticodon moiety and tRNA^Pyl^ can be engineered to read any other codons than UAG without impairing aminoacylation [7,8,9]. Thus, multiple PylRS variants were recently developed to genetically encode more than 100 synthetic amino acids [2,4] including α-hydroxy acids [10,11,12,13].

PylRS derived from Methanosarcinaceae, which is a subgroup of archaea, consists of the N-terminal and C-terminal domains (NTD and CTD, respectively) while bacterial PylRS consists of two different polypeptides (PylSn and PylSc). These are homologous to the NTD and CTD, respectively [14,15]. NTD is reportedly associated with the structural instability and aggregation-prone nature of the full-length PylRS in host cells [15,16,17], which probably underlies the poor productivity of proteins containing lysine derivatives. The genomic analysis of Methanomassiliicoccales, which is another archaeal group, recently revealed that PylRS from these organisms is homologous with CTD and PylSc while no PylSn equivalent is encoded in the genome [18,19,20]. Thus, Methanomassiliicoccales PylRS constitutes a new category of PylRS and the lack of NTD might be advantageous for the expression in heterologous host cells. *Candidatus* Methanomethylophilus alvus PylRS (CMaPylRS) belonging to this category reportedly incorporates lysine derivatives in *E. coli* cells as efficiently as Methanosarcinaceae PylRS [21].

In the present study, we compared CMaPylRS and PylRSs from the other groups in terms of the efficiency of UAG translation with various lysine derivatives. PylRSs of the first category were taken from *Methanosarcina* *mazei* and *Methanosarcina* *barkeri* (designated as MmPylRS and MbPylRS, respectively) while PylSc (DhPylSc) was taken from *Desulfitobacterium* *hafniense*. We found that the incorporation efficiency of CMaPylRS is much higher than those of the other PylRS species for various lysine derivatives. CMaPylRS was successfully modified in its amino acid specificity based on the sequence homology with the other molecules.

## 2. Results

### 2.1. E. coli Cell-Based Incorporation of Lysine Derivatives Using the Wild-Type Ca. M. alvus PylRS

**Figure 1:**
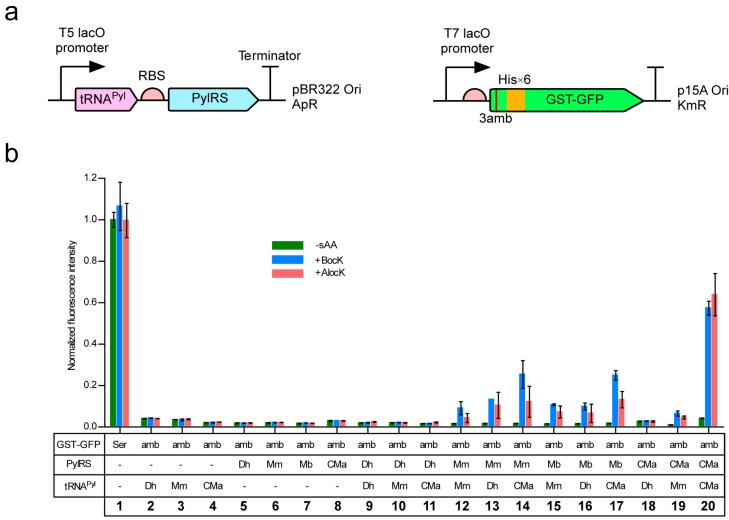
(**a**) Expression systems for PylRS, tRNA^Pyl^, and the GST-GFP reporter gene. “RBS”, “ApR”, and “KmR” represents the ribosome binding site, the ampicillin resistance, and the kanamycin resistance, respectively. (**b**) Cell-based fluorescence with the indicated combinations of PylRS and tRNA^Pyl^ relative to the average intensity for the “Ser” GST-GFP. “−sAA” represents the absence of BocLys (BocK) and AlocLys (AlocK). Three independent measurements were performed. The error bars indicate standard deviations.

Each of CMaPylRS, MmPylRS, MbPylRS, and DhPylSc was expressed from a lactose-inducible promoter in the *E. coli* BL21-Gold(DE3) strain together with one of tRNA^Pyl^ molecules from *D. hafniense*, *M. mazei*, and Ca. M. alvus (Figure 1a). Since the base sequence of tRNA^Pyl^ is identical between certain strains of *M. mazei* and *M. barkeri* and *M. mazei* tRNA^Pyl^ designated as Mm tRNA^Pyl^ was used as a common substrate for MmPylRS and MbPylRS. A rich growth medium was supplemented with either *N*^ε^-(*tert*-butoxycarbonyl)-l-lysine (BocLys) or *N*^ε^-allyloxycarbonyl-l-lysine (AlocLys) at a final concentration of 1 mM. Thus, the UAG stop codon was to be translated into either amino acid when the pyrrolysine pair was active enough. To observe UAG translation, we used a GST-GFP fusion protein in which glutathione *S*-transferase (GST) had an N-terminal hexahistidine tag and was fused with the “superfolder” green fluorescent protein (sfGFP) at the C-terminus. The serine codon at the third position from the N-terminus of the fusion protein was mutagenized to a UAG codon. The efficiency of UAG translation with the lysine derivatives was thus evaluated in terms of the intensity of the cell-based green fluorescence in comparison with that of the fluorescence from the GST-GFP fusion protein with no in-frame UAG (Figure 1b, lane 1). The cells lacking either the PylRS or tRNA^Pyl^ gene did not produce fluorescence (Figure 1b, lanes 2–8). DhPylSc paired with any of the tRNA^Pyl^ molecules failed to support the expression of the reporter (Figure 1b, lanes 9–11) and this observation is consistent with no activity or trace-level activity of DhPylSc without the PylSn partner, which was reported previously [14,22]. On the other hand, MmPylRS, MbPylRS, and CMaPylRS supported the expression of the reporter gene depending on the supplementation of the synthetic amino acids (Figure 1b, lanes 12–20). The cognate pairing between CMaPylRS and CMa tRNA^Pyl^ achieved a significantly higher efficiency than the Methanosarcinaceae cognate pairs. The relative fluorescence intensity was 0.6 for the CMa pair while the intensity was around 0.1 for the latter.

As for substrate specificities, CMaPylRS was found to hardly recognize Dh tRNA^Pyl^ and poorly recognize Mm tRNA^Pyl^ (Figure 1b, lanes 18 and 19) while the Methanosarcinaceae synthetases recognized all three tRNAs and exhibited higher efficiencies when paired with CMa tRNA^Pyl^ than paired with the cognate Mm tRNA (Figure 1b, lanes 12–17). CMaPylRS recognized BocLys and AlocLys with similar efficiencies (Figure 1b, lane 20) while the Methanosarcinaceae counterparts showed better activities with BocLys than AlocLys (Figure 1b, lanes 12–17). Thus, the CMa pair incorporated AlocLys more efficiently by factors of 14 and 9 than the Mm and Mb pairs, respectively.

### 2.2. Mass Spectromeric Analysis to Identify the Incorporated Amino Acid

**Figure 2:**
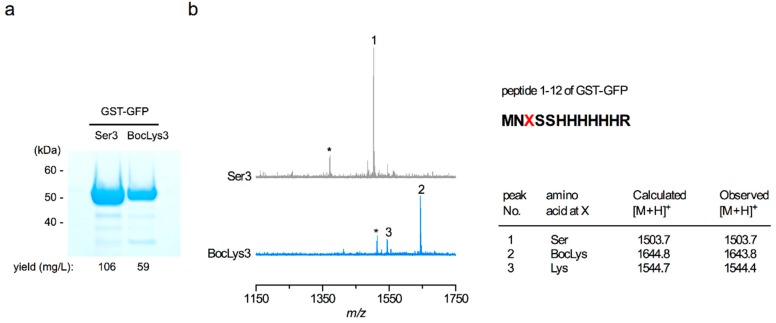
Analyses of the GST-GFP proteins with serine or amber codon at position 3. (**a**) These products were analyzed by SDS-polyacrylamide electrophoresis with Coomassie Brilliant Blue staining. (**b**) MALDI-ToF MS analyses of the trypsin-digested peptides of these products. The calculated and observed masses are tabulated in the table. The vertical axis indicates the signal intensity.

We obtained the GST-GFP amber product with a yield of 59 mg per one liter of cell culture using the CMa PylRS—tRNA^Pyl^ pair and BocLys while the yield of GST-GFP with no in-frame UAG was 106 mg/l (Figure 2a). In the mass spectrometric analyses of the trypsin-digested peptides of these two GST-GFP products, the signals corresponding to a peptide from residues 1 to 12 were observed (Figure 2b). The wild-type peptide contained Ser at position 3 while the corresponding peptide of the amber product contained BocLys and lysine at this position. This lysine was probably generated by the degradation of BocLys during the strong-acid treatment after the trypsin digestion. If lysine was mis-incorporated at position 3, the 12-residue peptide should have been cleaved after this position to generate a peptide from residues 4 to 12, which is a signal that was not observed. The peaks marked by the asterisks each correspond to a peptide from residues 2 to 12, which was probably generated by the removal of the N-terminal methionine. No peak corresponding to any peptide containing a canonical amino acid at position 3 in place of BocLys was detected. To confirm that the detected lysine at position 3 was derived from BocLys and not from mis-incorporation, we conducted ESI-TOF-MS analyses of the full-length GST-GFP products containing Ser and BocLys (Supplementary Figure S1). The difference in mass (140.0 Da) between the major peaks of these products corresponds to that in mass between Ser and BocLys and no peak corresponding to the product with lysine in place of BocLys was observed with a significant intensity. These results showed that the CMa PylRS—tRNA^Pyl^ pair exclusively incorporated the lysine derivative supplemented in the growth medium in response to the UAG amber codon.

### 2.3. E. coli Cell-Free Incorporation of a Lysine Derivative using the Wild-Type Ca. M. alvus PylRS

**Figure 3:**
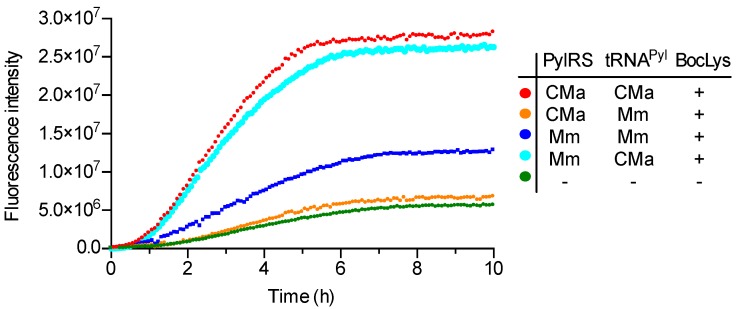
Cell-free UAG translation. The fluorescence intensity of the reaction mixture containing the indicated pair of PylRS and tRNA^Pyl^ was measured at 485 nm with five-minute intervals.

To explore the utility of the CMaPylRS—tRNA^Pyl^ pair in cell-free translation, an sfGFP variant gene with an in-frame UAG codon was translated in an *E. coli* reconstituted cell-free system [23]. Release factor 1, which terminates translation at the UAG codon, was omitted from the supplied components, to enhance the incorporation of amino acids at the UAG position. An increase in the intensity of the fluorescence of the reaction mixture was measured as an indicator of the incorporation of a lysine derivative at UAG. The separately prepared CMaPylRS, MmPylRS, CMa tRNA^Pyl^, and MmPylRS were added to the reaction mixture supplemented with BocLys at a final concentration of 1 mM and the fluorescence was measured for 10 hours (Figure 3). Although the background fluorescence level slowly increased in the absence of tRNA^Pyl^ and PylRS, a rapid increase in the fluorescence intensity was observed for the CMaPylRS—CMa tRNA^Pyl^ and MmPylRS—CMa tRNA^Pyl^ pairs. By contrast, the cognate Mm PylRS—Mm tRNA^Pyl^ pair supported a moderate increase while the CMaPylRS—Mm tRNA^Pyl^ pair supported no higher increase above the background level. Thus, the cognate CMa PylRS—tRNA^Pyl^ pair was significantly more efficient in incorporating the lysine derivative than the Mm pair. CMa tRNA^Pyl^ was found to be a better partner for Mm PylRS than its cognate tRNA while CMaPylRS hardly recognizes Mm tRNA^Pyl^. These finding were well consistent with the cell-based observations except that the MmPylRS—CMa tRNA^Pyl^ pair was apparently as efficient as the CMa cognate pair.

### 2.4. Activities of Ca. M. alvus tRNA^Pyl^ Variants in E. coli Cell-Based Translation

**Figure 4:**
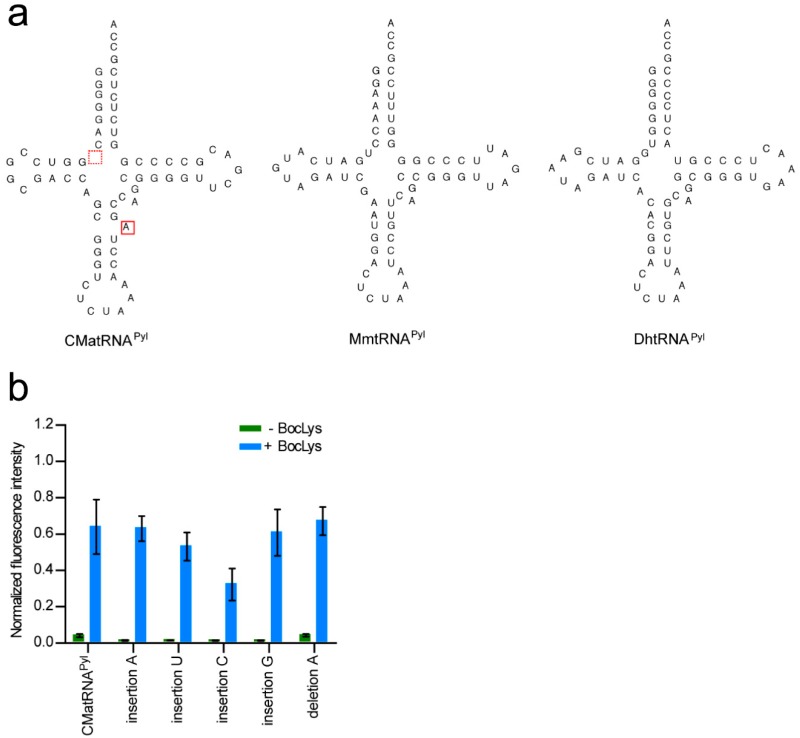
(**a**) CMa tRNA^Pyl^, Mm tRNA^Pyl^, and Dh tRNA^Pyl^ in the cloverleaf representation. (**b**) The intensity of the cell-based fluorescence was indicated for each of the modifications to CMa tRNA^Pyl^ relative to that of the fluorescence from the GST-GFP with no in-frame UAG. Three independent experiments were performed. The error bars indicate standard deviations.

The secondary structure of CMa tRNA^Pyl^ is different from those of the Mm and Dh tRNA^Pyl^ molecules with no connecting nucleotide between the acceptor and D stems and an additional, unpaired nucleotide in the anticodon stem (Figure 4a). The observation that CMaPylRS hardly recognized the Dh and Mm tRNA^Pyl^ molecules prompted us to determine if these features of CMa tRNA^Pyl^ are important for the recognition by CMaPylRS. The activities of CMa tRNA^Pyl^ variants were evaluated based on the cell-based synthesis of the reporter GST-GFP protein in the presence of CMaPylRS and BocLys (Figure 4b). The insertion of C or U between the acceptor and D stems reduced the level of the fluorescence moderately while the level was hardly changed by the insertion of A or G at the same position. The removal of the additional A in the anticodon stem did not change the fluorescence level. Thus, the idiosyncratic structural features of CMa tRNA^Pyl^ are not prerequisite for recognition by CMaPylRS.

### 2.5. E. coli Cell-Based Incorporation of Various Lysine Derivatives using the Wildtype and Variant CMaPylRS Molecules

**Scheme 1:**
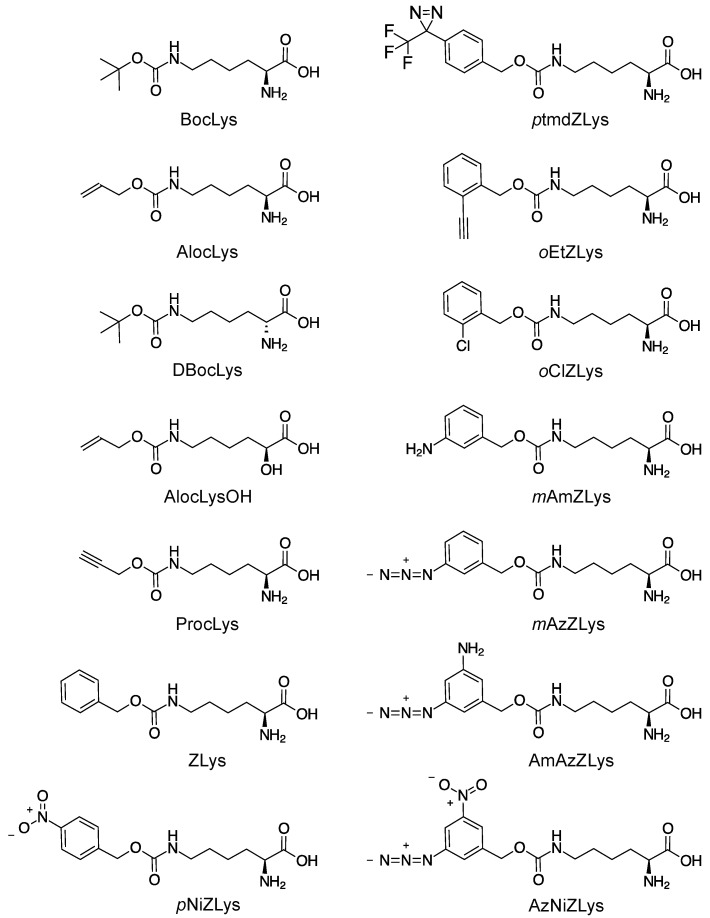
Chemical structures of lysine derivatives.

**Figure 5:**
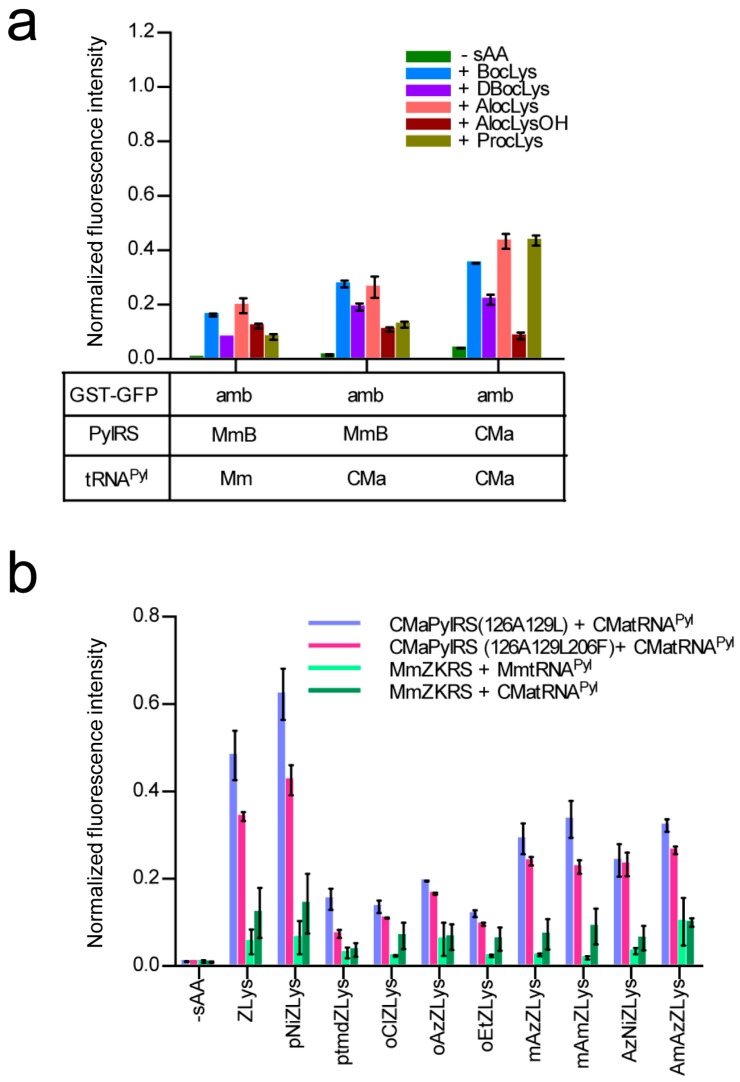
(**a**) The *E. coli* cell-based incorporation of smaller lysine derivatives by the wild-type CMa PylRS and MmBocLysRS2 represented by “MmB”. The error bars indicate standard deviations. (**b**) The *E. coli* cell-based incorporation of ZLys and its derivatives by CMa PylRS variants and MmZKRS. All of the indicated intensities of the fluorescence are relative to the cell-based fluorescence intensity of the GST-GFP with no in-frame UAG. Three independent experiments were performed. The error bars indicate standard deviations.

We examined the incorporation of more variations of lysine derivatives by CMaPylRS including the derivatives with small chemical groups attached to the *N*^ε^-carbonyl group and the derivatives of the bulky *N*^ε^-benzyloxycarbonyllysine (ZLys) (Scheme 1). The incorporation efficiency for the first group was compared between the wild-type CMaPylRS and a variant of MmPylRS, which improved with the activity of BocLys. This variant, MmBocLysRS2, contains a Tyr384Phe substitution and two auxiliary mutations (Arg61Lys and Gly131Glu) [24,25]. Based on the fluorescence from the reporter GST-GFP protein synthesized in the *E. coli* cell-based system, the CMa pair was found to be more efficient in incorporating BocLys, DBocLys, AlocLys, and ProcLys than the MmBocLysRS2—CMa tRNA^Pyl^ pair and MmBocLysRS2—Mm tRNA^Pyl^ pair (Figure 5a). By contrast, AlocLysOH, which is an α-hydroxy acid, was incorporated slightly more efficiently by the pairs involving MmBocLysRS2 than the CMa pair. It is likely that the amino acid substitutions in MmBoLysRS2 increased its affinity with the hydroxy acid [13].

**Figure 6:**
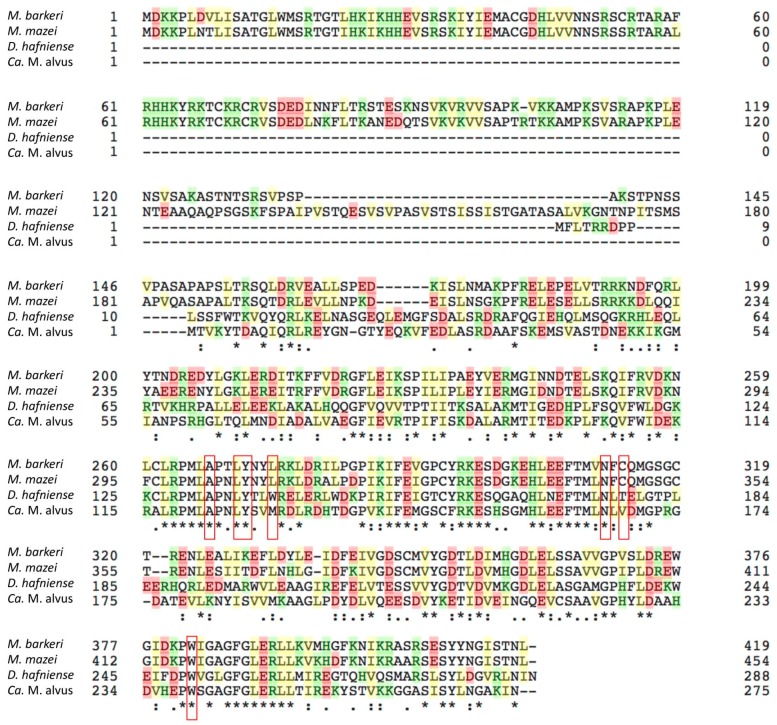
The alignment of the amino acid sequences of MmPylRS, MbPylRS, DhPylSc, and CMa PylRS. The residues at the pyrrolysine-binding pocket of MmPylRS and the corresponding residues in the other synthetases are indicated in the enclosures.

Next, we designed CMaPylRS variants able to recognize the bulky ZLys derivatives based on the amino-acid sequence alignment (Figure 6). Tyr306 and Tyr384 were previously replaced with Ala and Phe, respectively, in MmPylRS to create a variant (MmZKRS) that is able to recognize ZLys derivatives [24,26,27]. Tyr306 is located at the amino-acid binding site while Try384 is distant from the site. Leu309 is at the proximity of Try306 in the binding site and the replacement of this residue affects the substrate selection by PylRS [25]. These MmPylRS residues (Tyr306, Leu309, and Tyr384) correspond to Tyr126, Met129, and Tyr206, respectively, of CMaPylRS. We then created two CMaPylRS variants: one with the Tyr126Ala and Met129Leu substitutions and another with the Tyr126Ala, Met129Leu, and Tyr206Phe substitutions. Both variants, together with CMa tRNA^Pyl^, incorporated ZLys and the nine derivatives indicated in Scheme 1 much more efficiently than the MmZKRS—tRNA^Pyl^ pairs (Figure 5b). When compared between the CMaPylRS variants, CMaPylRS(126A129L) showed moderately higher incorporation rates than CMaPylRS(126A129L206F) for all of the derivatives.

## 3. Discussion

Methanosarcinaceae PylRS has been the “first choice” as a tool for site-specifically incorporating lysine derivatives into proteins. In the present study, we showed the advantage of the CMa PylRS—tRNA^Pyl^ pair in the production of proteins with such derivatives. Even though the efficiency of this pair for incorporating BocLys was reportedly moderately (1.2-fold) higher than that of the MmPylRS—tRNA^Pyl^ pair [21], we found that improvements in the efficiency can be much greater for various lysine derivatives. The improvement margin should vary under different experimental conditions. We used a strong promoter for expressing CMa PylRS in both the comparative analyses and the high-yield synthesis of proteins. The reporter protein containing BocLys at the UAG position was produced with a yield of 59 mg/L as opposed to 106 mg/L for the protein with no in-frame UAG. The proportion between these yields was well correlated with the relative intensity of the cell-based fluorescence (Figure 1b). We can then rely on the fluorescence data for various lysine derivatives to conclude that the CMa PylRS—tRNA^Pyl^ pair would be more advantageous than the “first-choice” PylRS systems in the large-scale production of proteins variants. The advantage of the CMa pair was also demonstrated in cell-free translation.

The crystal structure of MmPylRS has been reported [28,29] and a sequence alignment shows that Ala122, Leu125, Tyr126, Asn166, and Trp239 (in accordance with the numbering in CMaPylRS) at the pyrrolysine-binding pocket are conserved among MmPylRS, MbPylRS, DhPylSc, and CMaPylRS, which suggests that the substrate recognition manner is similar between these molecules. The engineering to expand the amino acid specificity of Methanosarcinaceae PylRS has involved the substitutions of Tyr306, Leu309, and Tyr384 (in accordance with the numbering in MmPylRS) [24,25,26,27,30,31,32,33,34]. Our CMaPylRs variants have replacements of the corresponding residues (Tyr126, Met129, and Tyr206, respectively). The effect of the Tyr126Ala substitution was probably enlarging the binding pocket to enable the recognition of the bulky ZLys derivatives with substituents on the benzoyl group. Although these amino acid residues were previously replaced to create the CMaPylRS variants recognizing ZLys and BCNK (a bicyclononyne-containing lysine) [21], our variants were distinct from them, which makes a variety of ZLys derivatives with useful chemical groups available in expanded codes. In addition, we showed that the wild-type CMaPylRS can efficiently incorporate not only BocLys but also various lysine derivatives with similar sizes into proteins. Our results, together with the previous report, showed the design-ability of CMaPylRS based on the accumulated knowledge of Methanosarcinaceae PylRS. Nevertheless, a structural study would provide a precise picture of the amino acid recognition by CMaPylRS and facilitate engineering to fully exploit the advantage of this synthetase.

The observation that CMaPylRS hardly recognizes Mm tRNA^Pyl^ previously led to the invention of two mutually orthogonal PylRS—tRNA^Pyl^ pairs [21]. Since CMa tRNA^Pyl^ assumes a distinct secondary structure from those of Mm and Dh tRNA^Pyl^ molecules [6], we assumed that this idiosyncrasy was an important factor in the recognition by CMaPylRS and lay behind the inability of this synthetase to recognize the noncognate tRNAs. However, the mutagenesis study of CMa tRNA^Pyl^ excluded this possibility. Being recognized by Methanosarcinaceae PylRS, CMa tRNA^Pyl^ should take an overall structure similar to that of Mm tRNA^Pyl^ despite the idiosyncrasies in the secondary structure. Therefore, CMaPylRS might recognize particular nucleotides that do not exist in the other tRNAs. It also remained to be addressed why CMa tRNA^Pyl^ more efficiently incorporates lysine derivatives than the cognate Mm tRNA^Pyl^ when paired with Methanosarcinaceae PylRS. This phenomenon was observed in both cell-based and cell-free systems. A systematic mutagenesis of CMa tRNA^Pyl^ would answer these questions.

## 4. Materials and Methods

### 4.1. Amino Acids

BocLys, AlocLys, DBocLys, *o*ClZLys, and *o*NiZLys were purchased from Bachem. ZLys was from Watanabe Chemical Industries (Hiroshima, Japan). AlocLysOH, *p*tmdZLys, *m*AmZLys, *o*AzZLys, *o*EtZLys, AmAzZLys, and AzNiZLys were from Shinsei Chemical Company (Osaka, Japan). ProcLys was from Synchem.

### 4.2. Plasmids

The gene coding for MmPylRS was obtained from the *M. mazei* strain JCM9314 and the rare glycine at position 444 was changed to Glu. Codon usage was not optimized for *E. coli* expression. The gene coding for MmBocLysRS2 was obtained from the plasmid described previously [25]. The CMaPylRS and DhPylRS genes were commercially synthesized. Each gene was fused with a DNA fragment carrying the *LacI* gene and T5/LacO promoter from pCDF-Mm2 [27], which is the ampicillin-resistance gene, a pBR322 replication origin, and the *rrnB* terminator from the pBAD vector (Invitrogen, Thermo Fisher Scientific K. K., Tokyo, Japan). The plasmids harboring PylRS gene were amplified by inverse PCR using primers with two split tRNA^Pyl^ sequences that have an overlap of 15 bases. PCR products were self-ligated using a NEBuilder HiFi DNA Assembly Master Mix reagent (New England BioLabs Japan, Tokyo, Japan) to create a series of pBT5 plasmids bearing different combinations of the PylRS and tRNA^Pyl^ genes under the control of the T5/LacO promoter. The PylRS gene was then removed by inverse PCR and self-ligation when a construct containing only a tRNA^Pyl^ gene was necessary. Mutagenesis to tRNA^Pyl^ or PylRS genes were conducted using a PrimeSTAR mutagenesis kit (Takara Bio Inc., Shiga, Japan). To create the reporter plasmid, the commercially synthesized DNA fragment carrying the T7/LacO promoter, a hexahistidine sequence, the glutathione *S*-transferase, superfolder green fluorescence protein genes, and the T7 terminator in this order was cloned into the pACYC177 vector (Nippon gene, Tokyo, Japan) to create pACYC-GST-GFP. An amber mutation was introduced in place of Ser3 just before the hexahistidine sequence using a PrimeSTAR mutagenesis kit to create pACYC-GST-GFP(amb3). For the biosynthesis and purification of PylRS, its gene was cloned in a derivative of the pET21b(+) vector (Novagen, Merck Millipore, Burlington, MA, USA) in which the T7 epitope tag was replaced by the sequence of a small ubiquitin-like modifier protein (SUMO), which was N-terminally tagged with a hexahistidine. The resulting plasmids were pET-SUMO-CMaPylRS and pET-SUMO-MmPylRS for CMaPylRS and MmPylRS, respectively.

### 4.3. Fluorescence Measurements of the E. coli Cell Culture

*E. coli* BL21-Gold(DE3) cells (Agilent Technologies K. K., Tokyo, Japan) were transformed with pACYC-GST-GFP (amb3) and a pBT5 plasmid or with pACYC-GST-GFP and pBR322 (Toyobo, Osaka, Japan), which is a control plasmid. The transformed cells were cultured at 25 °C for 24 h in the YT auto-induction medium [25] of 0.2 or 2 mL, which was supplemented with a lysine derivative at a final concentration of 1 mM. A 10-μL aliquot of the culture was then mixed with 190 μL of PBS in a 96-well clear-bottom black microplate. The fluorescence was measured using a SpectraMAX i3 plate reader (Molecular Devices Japan, Tokyo, Japan) with the excitation at 485 nm and the emission at 510 nm. The measured fluorescence intensity was normalized for the optical density at 600 nm.

### 4.4. Mass Spectrometry

The GST-GFP products were synthesized in *E. coli* BL21-Gold (DE3) transformed with the pBT5 plasmid expressing the CMa PylRS—tRNA^Pyl^ pair or pBR322 and it was cultured in a 5-mL YT auto-induction medium supplemented with BocLys. The cells were harvested by centrifugation and then lysed using the Bugbuster Master Mix reagent (Merck Millipore, Burlington, MA, USA). The supernatant of the centrifuged lysate was subjected to purification using a GST SpinTrap column (GE Healthcare Japan, Tokyo, Japan). The purified GST-GFP was analyzed by SDS-PAGE and stained with the SimplyBlue SafeStain reagent (Life Technologies Japan, Tokyo, Japan). A 25-μg aliquot of the GST-GFP was digested by Trypsin/Lys-C Mix Mass Spec grade (400 ng) (Promega K. K, Tokyo, Japan) at 37 °C overnight. The digested sample was applied to a His SpinTrap TALON column (GE Healthcare) and eluted with a 4% acetonitrile solution containing 0.1% trifluoroacetic acid (TFA). After evaporating the acetonitrile, mass spectrometry was performed commercially. ESI-TOF-MS analyses of the full-length products were commercially performed by a Mass Spectrometry Service, Research Resources Division, RIKEN Center for Brain Science (FUJI FILM Wako Pure Chemical Corporation, Osaka, Japan).

### 4.5. In Vitro Synthesis of tRNA^Pyl^

A template DNA for in vitro transcription of tRNA^Pyl^ was amplified from the pBT5 vectors containing tRNA^Pyl^ genes with an addition of the T7 promoter sequence to the 5′ end. The transcription was performed at 37 °C overnight in a 5-mL reaction [70 mM HEPES-K (pH 7.8), 16 mM MgCl_2_, 2 mM spermidine, 40 mM KCl, 5 mM dithiothreitol (DTT), 50 μg/mL bovine serum albumin, each 4 mM NTPs, 20 mM GMP, 1 unit/mL inorganic pyrophosphatase from yeast (Sigma-Aldrich Japan, Tokyo, Japan), 40 units/mL RNasein Plus Ribonuclease Inhibitor (Promega), and 7 μg/mL template DNA]. After the transcription reaction, RQ RNase-Free DNase (Promega) was added at a final concentration of 7 units/mL and incubated at 37 °C for 30 min. Transcribed tRNA was precipitated with isopropanol and then dried. The dried pellet was dissolved in 1.5 mL of 20 mM HEPES-Na buffer (pH 7.3) containing 5 mM MgCl_2_. The solution was heated at 80 °C for 10 min and then cooled to room temperature for renaturing tRNA. tRNA was then purified by anion exchange chromatography on a Protein-Pak Hi Res Q column (Nihon Waters K. K., Tokyo, Japan) using an ACQUITY UPLC H-Class Bio (Nihon Waters) at 25 °C with an 8:2 mixture of buffer A [20 mM HEPES-Na (pH 7.3) and 20 mM NaCl] and buffer B [20 mM HEPES-Na (pH 7.3) and 1 M NaCl] as the initial buffer. The separation was performed at a flow rate of 0.4 mL/min with a gradient of the proportion of buffer B from 20% to 100% in the mixture of buffer A and B over 3 min and then 100% B for 1 min monitored at 254 nm. The fraction containing tRNA was concentrated using an Amicon Ultra-4 10K filter unit (Merck Millipore). The concentration of tRNA in the obtained solution was determined by measuring the absorbance at 280 nm.

### 4.6. Purification of PylRS

To biosynthesize CMaPylRS, *E. coli* BL21-Gold (DE3) cells were transformed with the pET-SUMO-CMaPylRS plasmid. The transformed cells were cultured in a 50-mL 2×YT auto-induction medium at 20 °C for 2 days. The cells were harvested by centrifugation and then lysed using the Bugbuster Master Mix reagent. The supernatant of the centrifuged lysate was mixed with cOmplete His-Tag Purification resin (Roche Diagnostics K. K., Tokyo, Japan), which was then washed in 50 mM HEPES-Na buffer (pH 7.3) containing 500 mM NaCl, 30 mM KCl, 40 mM MgCl_2_, and 0.5 mM imidazole. The CMaPylRS was eluted in the buffer containing 150 mM imidazole and then subjected to digestion with 0.3 units/mL of SUMO protease (LifeSensors, Malvern, PA, USA) at 30 °C for 5 h. The released tag and the proteins retaining the tag were removed by applying the digests and 10-fold diluted with 30 mM HEPES-Na (pH 7.3) to a HiTrap Heparin HP column (GE Healthcare). The flow-through fraction containing CMaPylRS was then collected, which was then followed by a buffer exchange using an Amicon Ultra-4 10K filter unit to a 30 mM HEPES-Na (pH 7.3) buffer containing 140 mM NaCl, 30 mM KCl, 30 mM MgCl_2_, and 2 mM DTT. To synthesize MmPylRS, *E. coli* BL21-Gold (DE3) cells transformed with the pET-SUMO-MmPylRS were cultured in a 500-mL 2×YT auto-induction medium at 18 °C for 1 day and, then, harvested by centrifugation and lysed by sonication in 50 mM HEPES-Na buffer (pH 7.3) containing 500 mM NaCl, 5 mM 2-mercaptoethanol, 25 mM imidazole, and Protease Inhibitor Cocktail (EDTA free) (Nakalai Tesque Inc., Kyoto, Japan). The lysate was applied to a HisTrap HP column, which was washed with the sonication buffer, and eluted with the sonication buffer containing 475 mM imidazole. The eluted fraction was dialyzed with 50 mM HEPES-Na buffer (pH 7.3) containing 200 mM NaCl and 1 mM DTT (buffer A) at 4 °C overnight. The dialyzed fraction was purified on the ÄKTA prime system with a HiTrap Heparin column (GE Healthcare) using buffer A and 50 mM HEPES-Na buffer (pH 7.3) containing 2 M NaCl and 1 mM DTT (buffer B) with a gradient from 40% to 60% B. The fraction containing SUMO-MmPylRS was dialyzed with 50 mM HEPES-Na buffer (pH 7.3) containing 250 mM NaCl. Additionally, 1 mM DTT (buffer C) added SUMO protease at 4 °C overnight. The solution was purified on the ÄKTA prime system with a HiTrap SP column (GE Healthcare) using buffer C and buffer B with a gradient from 40% to 60% B. The fraction containing digested MmPylRS was purified on the ÄKTAexplorer 10S system with a Superdex 200 HiLoad 16/60 column (GE Healthcare) using 50 mM HEPES-Na buffer (pH 7.3) containing 300 mM NaCl and 1 mM DTT. MmPylRS in the fraction was concentrated using an Amicon Ultra-4 10K filter unit. The concentration of the purified PylRS was estimated by a Qubit Protein Assay Kit (Thermo Fisher Scientific K. K., Tokyo, Japan).

### 4.7. Cell-Free Translation

A template DNA fragment for the expression of GFP was amplified by PCR from pCDF-sfGFP with the forward and reverse primers and with the T7 promoter and terminator sequences, respectively. The coupled transcription-translation reaction was performed using the PURExpress delta RF123 Kit (New England BioLabs). The kit components except for release factor 1 were mixed according to the manual. The RNasein Plus Ribonuclease Inhibitor, template DNA, BocLys, PylRS and tRNA^Pyl^ were added to the mixed solution at the final concentration of 0.8 units/μL, 0.01 μg/μL, 1 mM, 2 μM, and 10 μM, respectively. The reaction mixture (50 μL) was incubated in a 96-well black microplate at 30 °C for 10 h. The fluorescence of the reaction mixture at 510 nm was measured with 5-min intervals for 10 h using the SpectraMAX i3 plate reader with an excitation at 485 nm.

### 4.8. Sequence Alignment

The alignment of the amino acid sequences was performed using the Clustal Omega software [35].

## Supplementary Materials

The following are available online, Figure S1: ESI-MS spectra of the full-length GST-GFP reporters with Ser and BocLys (upper and lower panels, respectively).
