# Supplementary material for: Pyrrolysyl-tRNA Synthetase with a Unique Architecture Enhances the Availability of Lysine Derivatives in Synthetic Genetic Codes
Source: Molecules. 2018 Sep 26;23(10):2460. doi: 10.3390/molecules23102460 (PMC6222415; doi:10.3390/molecules23102460)
Supplement: Supplementary file 1 [file molecules-23-02460-s001.pdf]

Supplementary data (Yamaguchi *et al.*)

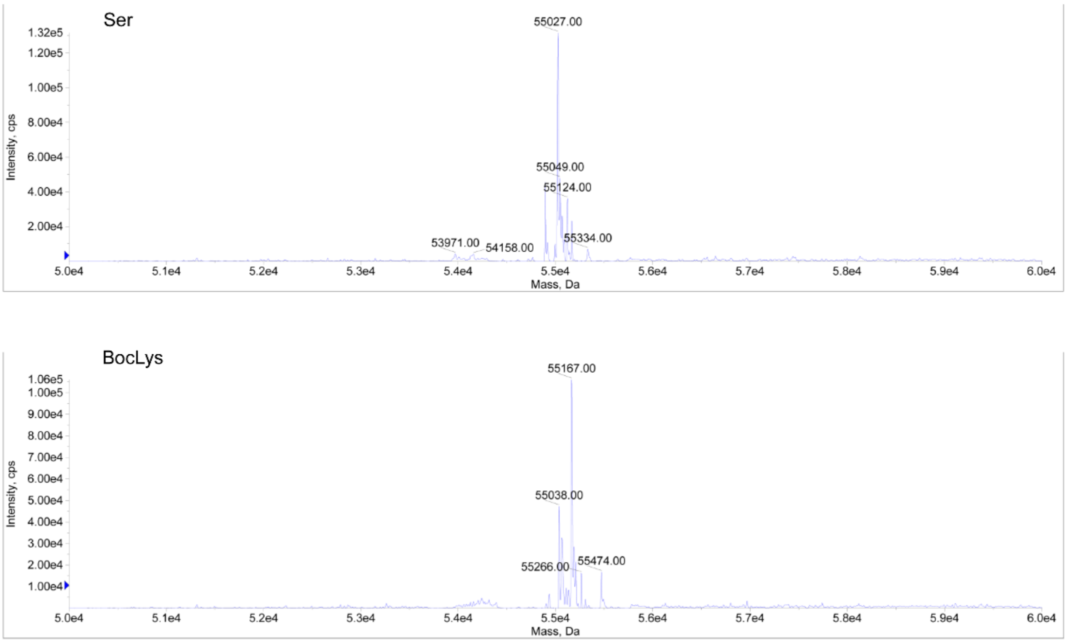

Supplementary Figure S1. ESI-MS spectra of the full-length GST-GFP reporters with Ser and BocLys (upper and lower panels, respectively).
